# Supplementary material for: Structural basis of TRPC4 regulation by calmodulin and pharmacological agents
Source: eLife. 2020 Nov 25;9:e60603. doi: 10.7554/eLife.60603 (PMC7735759; doi:10.7554/eLife.60603)
Supplement: Supplementary file 1. [file elife-60603-supp1.docx]

**Synthesis of GFB-8749**

**4,5-dichloro-2-(oxan-2-yl)-2,3-dihydropyridazin-3-one**

To a stirred solution of 4,5-dichloro-2,3-dihydropyridazin-3-one (50 g, 303 mmol, 1 equiv.) and 3,4-dihydro-2H-pyran (203.9 g, 2424 mmol, 8 equiv.) in THF was added 4-methylbenzene-1-sulfonic acid (10.4 g, 61 mmol, 0.2 equiv.) in portions at room temperature under nitrogen atmosphere. The resulting mixture was stirred for 2 days at 70 ^o^C under nitrogen atmosphere. The mixture was allowed to cool to room temperature. The resulting mixture was concentrated under reduced pressure. The residue was dissolved in water (500 mL). The resulting mixture was extracted with EtOAc (3 x 200mL). The combined organic layers were washed with water (3x200 mL), dried over anhydrous Na_2_SO_4_. After filtration, the filtrate was concentrated under reduced pressure. The residue was purified by silica chromatography, eluted with petroleum ether to afford 4,5-dichloro-2-(oxan-2-yl)-2,3-dihydropyridazin-3-one (57 g, 75.5%) as a light yellow solid.

**4-chloro-2-(oxan-2-yl)-5-(3-oxopiperazm-l-yl )-2, 3-dinydropyrutazm-3-one.**

To a stirred solution of 4,5-dichloro-2-(oxan-2-yl)-2,3-dihydropyridazin-3-one (47 g, 188 mmol, 1 equiv.) and piperazin-2-one (28.3 g, 283 mmol, 1.5 equiv.) in DMA was added DIEA (48.8 g, 377 mmol, 2 equiv.) in portions at room temperature under nitrogen atmosphere. The resulting mixture was stirred for 2 days at 110 ^o^C under nitrogen atmosphere and then concentrated under reduced pressure. The residue was dissolved in water (l L). The resulting mixture was extracted with DCM (2 x 500 mL). The combined organic layers were washed with water (2x300 mL), dried over anhydrous Na_2_SO_4_. After filtration, the filtrate was concentrated under reduced pressure. The residue was purified by silica gel column chromatography, eluted with DCM / MeOH (200:1 to 40:1) to afford 4-chloro- 2-(oxan-2-yl)-5-(3-oxopiperazin-1-yl)-2,3-dihydropyridazin-3-one (40 g, 68%) as an off white solid.

**4-chloro-5-[4-[(4,4-difluorocyclohexyl)methyl]-3-oxopiperazin-1-yl]-2-(oxan-2-yl)-2,3-dihydropyridazin-3-one**

To a stirred solution of 4-chloro-2-(oxan-2-yl)-5-(3-oxopiperazin-1-yl)-2,3-dihydropyridazin-3-one (300 mg, 0.96 mmol, 1 equiv) in DMF (6 mL) were added K_2_CO_3_ (398 mg, 2.88 mmol, 3 equiv) and 4-(bromomethyl)-1,1-difluorocyclohexane (613 mg, 2.88 mmol, 3 equiv) at rt. The reaction was stirred for 16 h at 100 ^o^C. The mixture was allowed to cool down to room temperature. The reaction was quenched with water (100 mL) at room temperature and extracted with EtOAc (3 x 500 mL). The combined organic layers were washed with brine (1x500 mL), dried over anhydrous Na_2_SO_4_. After filtration, the filtrate was concentrated under reduced pressure. The residue was purified by Prep-TLC (PE/EtOAc 80:1) to afford 4-chloro-5-[4-[(4,4-difluorocyclohexyl)methyl]-3-oxopiperazin-1-yl]-2-(oxan-2-yl)-2,3-dihydropyridazin-3-one(176 mg, 41%) as a yellow oil. LRMS (ESI) *m/z*: [M+H]^+^ calcd for C_20_H_28_ClF_2_N_2_O_3_ 445.17; found 445.15. Purity 95%.

**4-chloro-5-[4-[(4,4-difluorocyclohexyl)methyl]-3-oxopiperazin-1-yl]-2,3-dihydropyridazin-3-one (GFB-8749)**

To a stirred solution of 4-chloro-5-[4-[(4,4-difluorocyclohexyl)methyl]-3-oxopiperazin-1-yl]-2-(oxan-2-yl)-2,3-dihydropyridazin-3-one (100 mg, 0.22 mmol, 1 equiv) in DCM (10 mL) was added TFA (4 mL, 53.85 mmol, 240 equiv) in portions at room temperature. The resulting mixture was stirred for 16 h at rt. The resulting mixture was concentrated under reduced pressure. The residue was basified to pH =10 with saturated Na_2_CO_3_ (aq.) and extracted with EtOAc (3 x 100 mL). The combined organic layers were washed with brine (2 x 100 mL), dried over anhydrous Na_2_SO_4_. After filtration, the filtrate was concentrated under reduced pressure. The residue was purified by Prep-HPLC with the following conditions (Column: XBridge Shield RP18 OBD Column 30*150mm, 5um; Mobile Phase A:Water (10 mmol/L NH_4_HCO_3_), Mobile Phase B: ACN; Flow rate: 60 mL/min; Gradient: 10% B to 38% B in 7 min; 254 nm; Rt: 6.05 min) to afford 4-chloro-5-[4-[(4,4-difluorocyclohexyl)methyl]-3-oxopiperazin-1-yl]-2,3-dihydropyridazin-3-one (44 mg, 54 %) as a yellow semi-solid. ^1^H NMR (400 MHz, DMSO-*d*_6_) chemical shifts δ 12.97 (s, 1H), 7.87 (s, 1H), 4.10 (s, 2H), 3.71 (t, *J* = 5.3 Hz, 2H), 3.35 (s, 2H), 3.27 (s, 2H), 2.00 (s, 2H), 1.87 - 1.74 (m, 5H), 1.27 - 1.15 (m, 2H). LRMS (ESI) *m/z*: [M+H]^+^ calcd for C_15_H_20_ClF_2_N_4_O_2_ 361.12; found 361.05. Purity 98%.
